# Green Extraction of *Hodgsonia heteroclita* Oilseed Cake Powder to Obtain Optimal Antioxidants and Health Benefits

**DOI:** 10.3390/foods12234281

**Published:** 2023-11-27

**Authors:** Woorawee Inthachat, Sirinapa Thangsiri, Chanakan Khemthong, Nattira On-Nom, Chaowanee Chupeerach, Yuraporn Sahasakul, Piya Temviriyanukul, Uthaiwan Suttisansanee

**Affiliations:** Food and Nutrition Academic and Research Cluster, Institute of Nutrition, Mahidol University, Salaya, Phuttamonthon, Nakhon Pathom 73170, Thailand; woorawee.int@mahidol.ac.th (W.I.); sirinapa.tha@mahidol.ac.th (S.T.); chanakan.khe@mahidol.ac.th (C.K.); nattira.onn@mahidol.ac.th (N.O.-N.); chaowanee.chu@mahidol.ac.th (C.C.); yuraporn.sah@mahidol.edu (Y.S.); piya.tem@mahidol.ac.th (P.T.)

**Keywords:** agricultural waste, enzyme inhibition, genotoxicity, phenolics, response surface methodology, sustainability

## Abstract

Most biowaste produced by domestic food preparation and food processing has no value, is difficult to manage, and is detrimental to the environment. Oil extraction from *Hodgsonia heteroclita* seeds produces large amounts of oilseed cake powder (OCP) as biowaste. The extraction of residual phytochemicals using simple and eco-friendly methods can increase the economic utility of OCP. This study optimized the extraction process for *Hodgsonia heteroclita* OCP using a Box–Behnken design and response surface methodology. The optimized extraction condition was 30 °C for 5 h in 50% (*v*/*v*) ethanol, giving a total phenolic content (TPC) of 414.23 mg of gallic acid equivalent/100 g dry weight (DW). Phytochemical profiles of OCP using liquid chromatography-electrospray ionization tandem mass spectrometry (LC-ECI-MS/MS) identified 4-hydroxybenzoic acid and ferulic acid as the major compounds. Antioxidant activities and enzyme inhibitory activities toward the major enzymes involved in obesity (lipase), diabetes (α-amylase, α-glucosidase, and dipeptidyl peptidase IV (DPP IV)), Alzheimer’s disease (acetylcholinesterase (AChE), butyrylcholinesterase (BChE), and β-secretase-1 (BACE-1)), hypertension (angiotensin-converting enzyme, ACE), and genotoxicity were also investigated. Results showed that *H. heteroclita* OCP possessed antioxidant activity and potential inhibitory activities against BACE-1 and ACE, while also being genome-safe. A simple extraction method for *H. heteroclita* OCP was developed, demonstrating the enhanced value of its phytochemical and health-promoting qualities.

## 1. Introduction

*Hodgsonia heteroclita* subsp. *Indochinensis* W.J.de Wlide and Duyfjes, or Ma-King in Thai, is well known for its health benefits and high oil quality. This plant belongs to the Cucurbitaceae family and is distributed throughout southern Asia, including portions of Cambodia, Laos, and Thailand [1]. *H. heteroclita* seeds are rich in fat (32.5–33.5%), protein (26.7–27.6%), dietary fiber (13%), and vitamin E, especially δ-tocopherol and phytochemicals [2,3]. *H. heteroclita* seeds cultivated in the northern part of Thailand contain saturated fatty acids (mainly palmitic acid and stearic acid) at 35.67% and unsaturated fatty acids at 48.22%, with high amounts of Omega-6 (47,846 mg/100 g) and Omega-9 (11,362 mg/100 g), while low Omega-3 (57 mg/100 g) and no trans fats were observed [2]. Omega-3, -6, and -9 are considered potential ingredients for functional food industries, with the addition of several food supplements to reduce ailments such as inflammation and coronary heart disease [4,5], suggesting that high-quality oils from *H. heteroclita* seeds have health benefits. The rising demand for this oil has led to an increase in cultivation and wild harvesting. In Thailand, *H. heteroclita* is classified as an endangered species by the Plant Genetic Conservation Project under the initiative of Her Royal Highness Princess Maha Chakri Sirindhorn (RSPG). A substantial quantity of oilseed cake powder (OCP) is considered a biowaste from oil manufacturing, which is difficult to manage, environmentally unfriendly, and generally utilized as a low-cost fertilizer. However, phytochemicals have been reported in *H. heteroclita* fruits, including salicylic acid, caffeic acid, protocatechuic acid, and ferulic acid [6]. These phytochemical compounds have been widely explored for health benefits such as antioxidants, anti-inflammation, and anti-non-communicable diseases (NCDs) as well as cosmetics [7,8,9,10,11,12], thereby adding value to OCP by-products from *H. heteroclita* oil production. Therefore, this study aimed to optimize a green approach to achieve the extraction of high phytochemical contents from *H. heteroclita* OCP using a Box–Behnken design (BBD) and response surface methodology (RSM).

The idea of green extraction was first proposed by Chemat et al. based on procedures that reduce energy consumption by using alternative eco-friendly solvents and renewable natural resources to ensure safe, high-quality extracts [13]. In this study, BBD and RSM were used to support the green extraction of *H. heteroclita* seeds. BBD was used to reduce the number of experimental runs using varied experimental circumstances, thereby saving time and budget while maximizing the performance of the data obtained. RSM was utilized to forecast the optimal extraction condition based on the relationship between the input factors of interest. BBD is a type of RSM used to calculate the necessary number of experimental runs based on the number of factors of interest by predicting the interaction between the factors, or, in this case, extraction parameters [14].

Several parameters, including type of solvent, extraction time, and extraction temperature, have been considered in determining the extraction efficiency of phytochemicals. Ethanol is the solvent of choice in terms of environmentally friendly extraction due to its low toxicity compared to other alcohols (such as methanol) and organic solvents (such as hexane and dichloromethane), as well as its manageability, widespread use in the nutraceutical and food industries, and, most importantly, biodegradability [15,16,17]. A combination of ethanol and water improved extraction efficiency because the water assisted in plant cell expansion and breakage [18]. Direct data regarding the influence of extraction time and temperature on the extraction efficiency of *H. heteroclita* OCPs are lacking, but for pumpkin seeds (*Cucurbita moschata*), a member of the same family as *H. heteroclita*, extraction efficiency gradually increased as extraction time and temperature increased, while conversely, high extraction time and temperature reduced extraction efficiency [19]. Ahamad et al. reported that extraction time and temperature contributed to the extraction efficiency of charantin, a bioactive phytochemical from *Momordica charantia* (Cucurbitaceae) with anti-glycemic properties [20], implying that both extraction time and temperature contribute to the extraction efficiency of plants in the Cucurbitaceae family.

Three *H. heteroclita* oilseed extraction techniques were previously investigated, including (i) no pretreatment method, (ii) heat pretreatment, and (iii) heat and enzymatic pretreatment. Results suggested that heat and enzymatic pretreatment resulted in high oil quality [2]. Thus, OCP prepared using the heat and enzymatic pretreatment method was selected to optimize extraction using a simple procedure for repeatability purposes. As previously stated, ethanol concentration, extraction time, and temperature were included as extraction parameters. Under the optimal extraction condition, the three OCP extracts were further evaluated regarding their phytochemical contents, antioxidant activities, enzyme inhibitory activities against the key enzymes relevant to obesity (lipase), type II diabetes (α-amylase, α-glucosidase, and dipeptidyl peptidase-IV), Alzheimer’s disease (cholinesterases and β-secretase), hypertension (angiotensin-converting enzyme), and genotoxicity. This is the first report on extraction conditions to optimize phenolics from OCPs as well as their health-related properties. Knowledge gained from this study can be used to support the bio-circular economy of food plants and add economic value to OCP.

## 2. Materials and Methods

### 2.1. Sample Preparation

The fruits of *H. heteroclita* were collected from Hui-Nam-Guen village, Mae-Jedi-Mai sub-district, Weing-Pa-Pao district, Chiang Rai Province, Thailand, in October 2021. The plant was identified according to a reliable reference [21], while its voucher specimen was assigned as PBM 005646 by Sireeruckhachati Nature Learning Park, Mahidol University, Nakhon Pathom, Thailand.

Fresh endosperm (seed) of *H. heteroclita* was separated from the fruit and ground using a grinder (Philips 600 W series, Philips Electronics Co., Ltd., Jakarta, Indonesia) into fine powder (particle size < 80 mesh). This sample was assigned as a fresh sample and used to prepare oilseed cakes using 3 different mechanical oil extraction conditions, including extraction with no pretreatment method (NP), heat pretreatment by drying at 55 °C until reaching 10% moisture content (HP), and heat and enzymatic pretreatments using 2.98% (*w*/*w*) enzyme loading, 48 °C of incubation temperature, and 76 min of incubation time (HEP) according to the previous methods without any modification [2]. The oilseed cakes were then dried for 6 h in a 60 °C hot air oven (Memmert, Eagle, WI, USA) before being ground into fine powders (particle size < 80 mesh) and stored at −20 °C for further analysis. The samples were assigned as no pretreatment oilseed cake powder (NP-OCP), heat pretreatment oilseed cake powder (HP-OCP), and heat and enzymatic pretreatment oilseed cake powder (HEP-OCP). The color and moisture contents of all samples are shown in Supplementary Table S1.

### 2.2. Optimization of Extraction Conditions Using Box–Behnken Design (BBD) and Response Surface Methodology (RSM)

Three factors (ethanol concentration ($X_{1}$), extraction temperature ($X_{2}$), and extraction time ($X_{3}$)) were included in the BBD, and three variable degrees for each factor were employed, as shown in Table 1. These variable degrees resulted in 15 randomized runs listed in Table 2. Thus, the *H. heteroclita* OCP was extracted following the conditions in Table 2, and the total phenolic contents for each fraction were assayed.

### 2.3. Analyses of Phenolic Profile and Total Phenolic Contents

The phenolic profile was determined using liquid chromatography-electrospray ionization tandem mass spectrometry (LC-ESI-MS/MS) with parameters and validations as previously reported without any modification [22,23]. To prepare the samples, fresh samples and all OCPs were extracted under the optimized extraction conditions (30 °C for 5 h in 50% (*v*/*v*) ethanol) before all solvents were removed using a rotary evaporator until dryness. The dry extracts were re-dissolved with 62.5% (*v*/*v*) methanol before injection into LC-ESI-MS/MS. The chromatograms of all extracts are presented in Supplementary Figure S1.

Utilizing the optimized extraction conditions (30 °C for 5 h in 50% (*v*/*v*) ethanol), the total phenolic contents (TPCs) of fresh and OCP extracts were determined using Folin–Ciocalteu phenol reagent according to a previously established protocol without any modification [24]. An assay was detected using a 96-well UV-visible microplate reader (Synergy™ HT series from BioTek Instruments, Inc., Winooski, VT, USA) and Gen 5 data analysis software (version 2.09). Gallic acid (0–200 µg/mL) was employed to generate a standard calibration curve. TPC was expressed as mg gallic acid equivalent (GAE)/100 g dry weight (DW).

### 2.4. Determination of Antioxidant Potentials

The antioxidant potentials of the fresh sample and OCPs extracted under the optimized extraction conditions were determined by 2,2-diphenyl-1-picrylhydrazyl (DPPH) radical scavenging, ferric ion-reducing antioxidant power (FRAP), and oxygen radical absorbance capacity (ORAC) assays as previously reported without any modification [25]. Different concentrations of Trolox were used to generate a standard calibration curve with a microplate reader. All assays used Trolox at different concentrations as a standard, and antioxidant activities were expressed as µmol of Trolox equivalent (µmol TE)/100 g DW.

### 2.5. In Vitro Enzyme Inhibitory Activities

Using optimized extraction conditions, fresh and OCP extracts were investigated regarding their inhibitory activities against the key enzymes relevant to obesity (lipase), diabetes (α-amylase, α-glucosidase, and dipeptidyl peptidase-IV (DPP-IV)), Alzheimer’s disease (acetylcholinesterase (AChE), butyrylcholinesterase (BChE), and β-secretase (BACE-1)), and hypertension (angiotensin-converting enzyme (ACE)) as previously reported without any modification [24]. Lipase, α-amylase, α-glucosidase, DPP-IV, AChE, and BChE inhibitory assays were kinetically detected, while BACE-1 and ACE inhibitory assays were endpoints measured using the microplate reader. The results were expressed as a percentage of inhibition (% inhibition) using the following equation:

(1) $$\%\;\mathrm{inhibition}\;=\left(1-\;\frac{B-b}{A-a}\right)\times \;100,$$

where *A* is an initial rate (per s) of a reaction with an enzyme but without an extract (control), *a* is an initial rate (per s) of a reaction without an enzyme and an extract (control blank), *B* is an initial rate (per s) of a reaction with an enzyme and an extract (sample), and *b* is an initial rate (per s) of a reaction with an extract but without an enzyme (sample blank).

### 2.6. Bacterial Reverse Mutation Test (Ames Test)

The Ames test, or bacterial reverse mutation test, was carried out using the Organization for Economic Co-operation and Development’s (OECD) guideline for testing of chemicals No. 471, ‘Bacterial Reverse Mutation Test’. The assay used five strains of *Salmonella typhimurium*, including TA98, TA100, TA102, TA1535, and TA1537, to detect various types of mutagens. To differentiate between direct and indirect mutagens, the experiments were divided into two types: those with and without S9 extract, which was purchased from Sigma-Aldrich (St. Louis, MO, USA). The number of revertant colonies, indicating the presence of DNA mutations, was counted and compared with the negative (dimethyl sulfoxide) and the positive controls. For experiments without S9 activation, the positive controls were 4-nitroquinoline-1-oxide (4NQO, 0.2 µg/plate for TA98), sodium azide (NaN_3_, 2.5 µg/plate for TA100 and 0.5 µg/plate for TA1535), mitomycin C (MMC, 0.5 µg/plate for TA102), and 9-aminoacridine (9-AA, 50 µg/plate for TA1537). For experiments with S9 activation, a positive control, 2-aminoanthracene (2-AA, 2 µg/plate), was used. The mutagenicity ratio (MR) was calculated from the average of the revertant number divided by the average of the negative control revertant number.

### 2.7. Statistical Analysis

All experiments were performed in triplicate (*n* = 3) and reported as mean ± standard deviation (SD). The Box–Behnken design, one-way analysis of variance (ANOVA), and graphical analysis of the obtained data were performed using the software Design-Expert (Stat-Ease Inc., Minneapolis, MN, USA). The ANOVA and Duncan’s multiple comparison were used to assess the difference between samples in experiments, with *p* < 0.05 indicating a statistically significant difference.

## 3. Results

### 3.1. Optimization of Extraction Conditions

#### 3.1.1. Model Fitting and Variance Analysis

The HEP-OCP was extracted based on Table 2. The TPCs extracted from HEP-OCP ranged from 133.24 to 427.55 mg GAE/100 g DW, with the highest from $X_{1}:X_{2}:X_{3}$ = 50% *v*/*v*:70 °C:4 h and the lowest from $X_{1}:X_{2}:X_{3}$ = 90% *v*/*v*:30 °C:4 h (Table 3).

ANOVA was used to analyze model fitting, linearity of the factors, quadratic influence of the independent variables, their interactions, and coefficients on the response of the extraction analysis, as shown in Table 4. The regression model with a significance level (*p*-value) of 0.0022 suggested that the constructed model well-fitted the data. Moreover, the R^2^ and its adjusted values (R^2^ adjusted) for TPC were 97.23% and 92.26%, respectively, and close to the maximum at 100%, indicating that the model could be used to predict TPC with high validity. In support of this, the *p*-value for lack of fit was not significant (*p* = 0.3436), indicating that the quadratic analysis was appropriate. Finally, a predicted second-order polynomial regression equation for TPC was generated.

(2) $$Y=573.648-2.159X_{1}-4.456X_{2}+49.383X_{3}+0.040X_{1}X_{2}+0.121X_{1}X_{3}-0.545X_{2}X_{3}-0.044X_{1}^{2}+0.044X_{2}^{2}-3.815X_{3}^{2}$$

where Y is the predicted TPC (mg GAE/g DW) and $X_{1},\;X_{2},$ and $X_{3}$ are the ethanol concentration (% *v*/*v*), temperature (°C), and time (h) of the independent variables, respectively. The prediction equation was used to calculate the TPC produced by the optimal extraction condition to analyze antioxidant content and enzyme activity as well as assess genotoxicity.

The ANOVA data in Table 4 demonstrated that only the ethanol concentration ($X_{1}$) contributed to TPC because the *p*-value of the monomial coefficient of $X_{1}$ was less than 0.05 (*p* < 0.0001). Other monomial coefficients, including $X_{2}$ and $X_{3}$, were higher than 0.05, indicating that the linear terms of extraction temperature and extraction time had no significant impact on the TPC extracted from HEP-OCP. No interactions between the independent variables, including $X_{1}X_{2},\;X_{1}X_{3},$ and $X_{2}X_{3}$, were observed since the *p*-values were greater than 0.05. Therefore, only the ethanol concentration played a significant role in the TPC extraction process from HEP-OCP.

**Figure 1:**
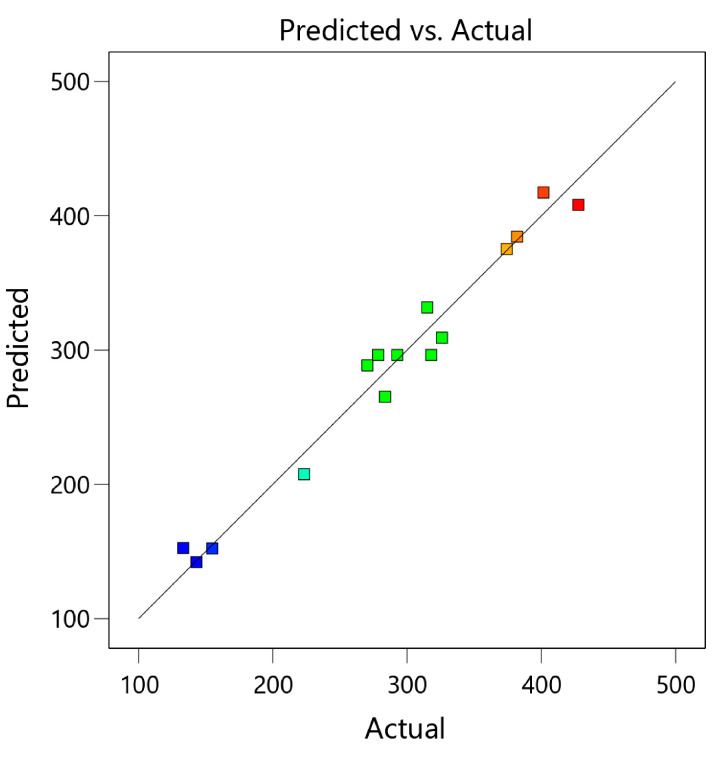
Experimentally measured total phenolic contents (TPCs) of *H. heteroclita* oilseed cake powder obtained from heat and enzymatic pretreatment (HEP-OCP) vs. predicted TPCs calculated from Equation (2). The different colors were corresponded to the color explained in Figure 2, in which the red color indicated high TPCs, while the blue color indicated low TPCs.

To test the validity of the prediction equation (Equation (2)), this equation was used to calculate the predicted TPCs compared with actual experiment data. Figure 1 shows that the correlation was at R^2^ = 0.9467, indicating the high validity of this equation.

#### 3.1.2. Response Surface Analysis (RSM)

**Figure 2:**
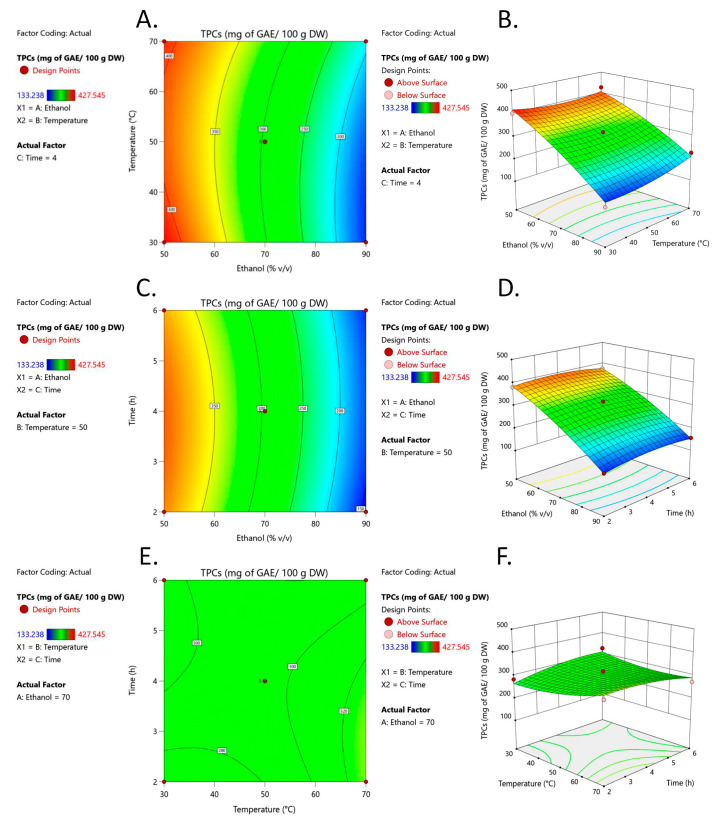
Response surface plots illustrating the interaction effects of temperature and ethanol concentration (**A**,**B**), time and ethanol concentration (**C**,**D**), and temperature and time (**E**,**F**) on total phenolic contents (TPCs) extracted from *H. heteroclita* oilseed cake powder obtained from heat and enzymatic pretreatment (HEP-OCP). Intermediate levels of TPCs are displayed as a green color, while high levels of TPCs are shown in the graph surface area as a red color and low levels as a blue color.

To study the interaction between the independent variables on TPC levels, contour and three-dimensional (3D) response surface plots were generated, as shown in Figure 2. A gradual increase (50–90% *v*/*v*) in ethanol concentration resulted in a reduction of TPC (Figure 2A–D). However, TPC was not significantly affected by extraction temperature or extraction time, as shown in Figure 2F, in which changes in extraction time and temperature had no significant effect on the response surface plots for TPC extraction. In support of this, neither extraction temperature nor extraction time contributed to TPC extraction from HEP-OCP (Figure 2E,F) because the surface did not change when both independent variables were changed, thereby confirming the ANOVA results in Table 4.

The optimized condition for the highest TPC extraction from HEP-OCP was analyzed by Design-Expert (Version 13, Stat-Ease Inc., Minneapolis, MN, USA) using data from Table 3. Results showed that the optimal extraction conditions were ethanol concentration:extraction temperature:extraction time at 50% *v*/*v*:30 °C:5.127 h, with desirability at 98.2%. The ethanol concentration, extraction temperature, and extraction time were then adjusted to 50%, 30 °C, and 5 h for reproducibility purposes. At this condition, the predicted TPC was 422.16 mg of GAE/100 g DW, while the experimental value was 413.96 mg of GAE/100 g DW.

### 3.2. Phenolic Profiles and Total Phenolic Contents

The phenolic profiles of the fresh sample and all OCPs were determined by LC-ESI-MS/MS analysis and 24 authentic phenolic standards (apigenin, caffeic acid, chlorogenic acid, *p*-coumaric acid, cinnamic acid, 3,4-dihydroxybenzoic acid, ferulic acid, (−)-epigallocatechin gallate, galangin, syringic acid, genistein, hesperidin, kaempferol, 4-hydroxybenzoic acid, rosmarinic acid, isorhamnetin, naringenin, luteolin, myricetin, quercetin, rutin, sinapic acid, gallic acid, and vanillic acid). Results indicated that the fresh sample and HP-OCP contained 4-hydroxybenzoic acid, caffeic acid, and ferulic acid, while NP-OCP and HEP-OCP contained only 4-hydroxybenzoic acid and ferulic acid (Table 5). Among all the phenolics detected in the fresh sample (100 g DW), ferulic acid was predominant (896.31 mg), followed by 4-hydroxybenzoic acid (727.68 mg) and caffeic acid (501.72 mg). A similar trend was observed in the OCPs, with ferulic acid being the highest, except for HEP-OCP, which contained predominantly 4-hydroxybenzoic acid, followed by ferulic acid. Interestingly, the phenolics detected in the fresh sample were higher than in OCPs (1.3–1.8-fold higher in 4-hydroxybenzoic acid, 1.4-fold higher in caffeic acid, and 1.4–3.0-fold higher in ferulic acid). The HP-OCP exhibited higher phenolics than the NP-OCP and HEP-OCP (1.4-fold higher in 4-hydroxybenzoic acid and 1.1–2.2-fold higher in ferulic acid, while caffeic acid was only detected in the HP-OCP).

Contrasting results were observed when TPC was analyzed by the spectrophotometric method utilizing the Folin–Ciocalteu phenol reagent (Table 5). All OCPs exhibited TPCs ranging from 117.00 to 413.96 mg GAE/100 g DW, 2.7–9.4-fold higher than the fresh sample (43.87 mg GAE/100 g DW). The HEP-OCP exhibited the highest TPC at 3.0–3.5-fold higher than the others.

### 3.3. Antioxidant Activities

The antioxidant potentials of the fresh sample and the OCPs were determined utilizing DPPH radical scavenging, FRAP, and ORAC assays, as shown in Table 6. The results indicated that the fresh samples possessed antioxidant activities with DPPH radical scavenging activities of 0.04 µmol TE/100 g DW, FRAP activities of 0.88 µmol TE/g DW, and ORAC activities of 9.65 µmol TE/g DW, while the OCPs exhibited antioxidant activities with DPPH radical scavenging activities of 0.10–0.56 µmol TE/100 g DW, FRAP activities of 2.14–7.78 µmol TE/g DW, and ORAC activities of 25.32–68.72 µmol TE/g DW. The antioxidant activities of the OCPs were higher than those of the fresh sample by 2.4–13.9-fold in DPPH radical scavenging activities, 2.4–8.8-fold in FRAP activities, and 2.6–7.1-fold in ORAC activities. The HEP-OCP exhibited higher antioxidant activities than the HP-OCP and NP-OCP, respectively (4.3–5.8-fold higher in DPPH radical scavenging activities, 2.7–3.6-fold higher in FRAP activities, and 2.1–2.7-fold higher in ORAC activities).

### 3.4. In Vitro Health-Promoting Activities

Inhibitory activities against the key enzymes relevant to obesity (lipase), diabetes (α-amylase, α-glucosidase, and DPP-IV), Alzheimer’s disease (AChE, BChE, and BACE-1), and hypertension (ACE) were investigated using the fresh sample and OCPs extracted under the optimal extraction condition, as shown in Table 7. Inhibition of lipase, a lipid-degrading enzyme, leads to retardation of fat absorption by the body, a key factor in controlling obesity from fat overconsumption. Using the final extract concentration of 20 mg/mL, only the fresh sample exhibited lipase inhibitory activity at 10.63% inhibition, while no detected activities were observed in the OCPs.

Contrasting results were observed in inhibitory assays against the key enzymes involved in controlling diabetes. Two carbohydrate-degrading enzymes (α-amylase and α-glucosidase) and the insulin secretion-stimulating enzyme (DPP-IV) were investigated. Using the final extract concentration of 20 mg/mL, only HEP-OCP exhibited α-amylase inhibitory activity (although only 3.72% inhibition was detected). Similar results were observed in the DPP-IV inhibitory assay, with only HEP-OCP exhibiting 16.66% inhibition. However, the fresh sample and all the OCPs exhibited α-glucosidase inhibitory activity (11.56% inhibition in the fresh sample and 9.42–15.81% inhibition in the OCPs). Among these, HEP-OCP exhibited the highest α-glucosidase inhibition, 1.4–1.7-fold higher than the others.

Two neurotransmitter-degrading enzymes (AChE and BChE) and the β-amyloid formation enzyme (BACE-1) were investigated to control Alzheimer’s disease. When using the final extract concentration of 20 mg/mL, no inhibitory activities against AChE and BChE were observed in all samples. Interestingly, high BACE-1 inhibitory activities were found in both the fresh sample (39.80% inhibition) and OCPs (65.40–76.60% inhibition) using the same extract concentration. All OCPs exhibited 1.6–1.9-fold higher BACE-1 inhibitory activities than the fresh sample, with HEP-OCP exhibiting 1.1–1.2-fold higher inhibitory activities than the other two OCPs.

In controlling hypertension, inhibition of ACE results in lower levels of the active vasoconstrictor, angiotensin II, leading to lower blood pressure. Using the final extract concentration of 20 mg/mL, ACE inhibitory activities were detected in both the fresh sample (25.77% inhibition) and OCPs (68.47–93.52% inhibition). All OCPs exhibited 2.7–3.6-fold higher ACE inhibitory activities than the fresh sample, with HEP-OCP exhibiting 1.2–1.4-fold higher ACE inhibitory activities than the other two.

### 3.5. Genotoxicity Assessment

The evaluation of safety is an essential requirement to promote the usage and future application of optimized HEP-OCP extract in the food industry. Thus, a genotoxicity analysis was performed as one aspect of food safety using the Ames test. This genotoxicity assay is used by the Organization for Economic Co-operation and Development (OECD) as a standard guideline. Five *S. typhimurium* strains, including TA98, TA100, TA102, TA1535, and TA1537, were used to detect several types of mutagens. Some mutagens (indirect mutagens) must be bioactivated by S9 extract before they become direct mutagens; hence, studies were conducted both with and without S9 extract. Table 8 demonstrates that bacteria incubated with the optimized HEP-OCP extract (10–2000 µg/plate) in the absence of S9 exhibited revertant colonies comparable to the negative control (DMSO), with MR of approximately 1.00, whereas all positive controls (4-NQO, NaN_3_, MMC, and 9-AA) exhibited a high number of revertant colonies with MR ≥ 2.00, indicating that the optimized HEP-OCP extract did not induce DNA mutations in all five strains. Results in Table 9 show that the number of revertant colonies of bacteria treated with the extract (10–2000 µg/plate) in the bioactivation condition (+S9) was also comparable to the negative control (DMSO), with MR values of about 1.00, while MR was greater than 2.00 in a positive control (2-AA). Taken together, the data from Table 8 and Table 9 suggested that the optimized HEP-OCP extract did not promote DNA mutations in the presence or absence of bioactivation and was therefore genome-safe.

## 4. Discussion

Agricultural and food processing wastes normally have low-value applications such as fertilizers or feed ingredients. Extracting bioactive compounds from biowaste has gained increasing attention in promoting the circular economy, following the zero-food waste concept [26,27,28]. *H. heteroclita* OCP, as a by-product from oilseed extraction, contains valuable bioactive compounds with health benefits. A green extraction method was optimized to achieve optimal TPC and investigate the phenolic profile, antioxidant potential, enzyme inhibition, and genotoxicity of the OCP extracts. Results indicated that only the ethanol concentration impacted TPC extraction from HEP-OCP, while extraction temperature and time had no significant effect. Under optimal extraction conditions, the highest TPC was detected in HEP-OCP, correlating with its antioxidant potential and enzyme inhibitory activity. In addition, HEP-OCP was found to be genome-safe.

Three parameters that influenced extraction efficiency were selected, including aqueous ethanol concentration (*v*/*v*), extraction time (h), and extraction temperature (°C). Only the ethanol concentration affected TPC extraction from *H. heteroclita* OCP, while extraction time and temperature were irrelevant (Table 4). Interestingly, ethanol concentration did not interact with either extraction time or temperature, implying that it was an important parameter for TPC extraction from *H. heteroclita* OCP. A 3D plot of the ethanol concentration (Figure 2) showed that an increase from 50 to 90% *v*/*v* resulted in decreased TPC, suggesting that most of the TPC presented in *H. heteroclita* OCPs was hydrophilic because 50% (*v*/*v*) aqueous ethanol has a polarity index (PI) of 7.1 while absolute ethanol has a PI of 5.2 [29]. Water supports plant cell expansion and breakage, leading to higher TPC in 50% (*v*/*v*) aqueous ethanol compared with 90% (*v*/*v*) aqueous ethanol [18]. Our findings were consistent with those of Sarkis et al., who stated that ethanol concentration contributed to TPC extraction efficiency from sesame seed cake through the same phenomenon. A gradual increase in ethanol concentration led to an increase in TPC [30]. However, TPC started to decline when the ethanol concentration became too high, with an optimal value of 68% (*v*/*v*). Temperature and time were included as extraction parameters because an increase in temperature increases the solubility of TPC, while increased extraction time leads to high penetration of TPC from the plant cells into solvents. Extraction temperature and time did not impact TPC extraction from *H. heteroclita* OCPs, as shown by the 3D plot (Figure 2). The combination of low ethanol concentration and reduced extraction temperature or low ethanol concentration and less extraction time resulted in high TPC, indicating that TPC concentration had already reached saturation. Thus, further temperature or time increases did not affect TPC yield. A study on seeds from the same family as *H. heteroclita*, *Cucurbita maxima*, revealed that the relationship between ethanol concentration and extraction duration had little impact on TPC extraction [31]. Even though 5 h extraction was considered a particularly long extraction period, prolonged extraction times were previously reported on *H. heteroclita* fruit. For example, (i) extraction time (18–24 h) and absolute methanol were used to extract TPCs from *H. heteroclita* fruits, and this resulted in TPCs of 1945 mg GAE/100 g DW [32], (ii) extraction time (24 h) and absolute methanol were used to extract TPCs from *H. heteroclita* fruits, and this resulted in TPCs of 800.95 GAE/100 g DW [1], (iii) extraction time (6 h) and 70% (*v*/*v*) methanol were used to extract TPCs from *H. heteroclita* rinds, and this resulted in TPCs of 1891 mg GAE/100 g DW [33], and extraction time (12 h) and 96% (*v*/*v*) ethanol were used to extract TPCs from oil seed cakes of camelina, linseed, and rapeseed, yielding TPCs of 12 to 450 mg GAE/100 g DW [34].

Using the optimal extraction condition (ethanol concentration: extraction temperature: extraction time = 50% (*v*/*v*): 30 °C: 5 h), the TPC of our OCPs (117–414 mg GAE/100 g DW, Table 5) concurred with previous reports on OCP prepared from other plant materials. An 80% (*v*/*v*) aqueous methanolic extract of flaxseed, sesame, mustard, nigella, and groundnut OCPs exhibited TPCs ranging from 138 to 512 mg GAE/100 g DW [35]. Previous research has reported TPCs of OCPs as per g fresh weight (FW) and, therefore, unsuitable for comparison with our results. However, OCPs normally have a low moisture content (<10%) [35], which can be compared with our results. An ethanolic extract of hemp OCP yielded a TPC of 351 mg GAE/100 g FW, while that of flax OCP extracted under the same solvent exhibited a TPC of 255 mg GAE/100 g FW [36]. Camelina, linseed, and rapeseed OCPs extracted with 96% (*v*/*v*) aqueous ethanol exhibited TPCs of 12 to 450 mg GAE/100 g FW [34], while 80% (*v*/*v*) aqueous ethanolic extracts of flax, milk thistle, hemp, safflower, poppy, rapeseed, pumpkin, and sunflower OCPs exhibited TPCs of 145 to 3044 mg GAE/100 g FW [37]. However, OCPs of hemp, pumpkin, and flax seed extracted by advanced technology subcritical water extraction exhibited TPCs of 3.26 to 3.56 mg GAE/100 g DW [38]. These results demonstrated that the cost-efficient and environmentally friendly extraction method proposed in this study efficiently extracted residual phytochemicals from *H. heteroclita* OCPs. Phenolic profiles analyzed by the HPLC technique indicated that types and quantities of phenolics depended on particular oilseeds [35,36]. Our results and previous reports [35,36] indicated that TPC was not correlated with phenolic quantities identified by the HPLC technique, suggesting that the types of phenolic standards used in the HPLC analysis might not cover all predominant phenolics in the plant samples.

Phenolics can act as effective antioxidants. Our results were in line with previous studies, suggesting that TPCs were highly correlated to antioxidant activities, whereby OCPs with high TPCs led to high antioxidant activities [39]. An 80% (*v*/*v*) aqueous methanolic extract of flaxseed, sesame, mustard, nigella, and groundnut OCPs exhibited DPPH radical scavenging activities ranging from 5.80 to 12.28 µmol TE/g DW [35], while 96% (*v*/*v*) aqueous ethanolic extracts of camelina, linseed, and rapeseed OCPs exhibited DPPH radical scavenging activities with half maximal effective concentration (EC_50_) values of 2.8 to 5.7 µg/mL [34]. An ethanolic extract of hemp, flax, and canola OCPs exhibited DPPH radical scavenging activities of 3.09 to 15.07% inhibition using an extract concentration of 147 µg/mL [36]. Flax, milk thistle, hemp, safflower, poppy, rapeseed, pumpkin, and sunflower OCPs extracted with 80% (*v*/*v*) aqueous ethanol exhibited DPPH values ranging from 0.37 to 11.37 mg ascorbic acid equivalent (AAE)/g FW [37], while ethanolic extracts of hemp, flax, and canola OCPs possessed FRAP activities ranging from 0.51 to 2.45 µmol Fe (II)/g FW [36]. Previous studies investigated the antioxidant potentials of OCPs from different plant materials using diverse antioxidant assays, standards, and calculation methods, thereby making the comparison of results difficult. However, despite these differences, the trend between TPC and antioxidant potentials, in which the sample with high TPC exhibited elevated antioxidant activity while the low TPC one exhibited low antioxidant activity, remained throughout these reports.

Other than being effective antioxidants, some phenolics also act as strong inhibitors of the key enzymes relevant to some NCDs. Our results indicated that OCPs strongly inhibited BACE-1 and ACE (>50% inhibition), possibly due to the biological function of predominant 4-hydroxybenzoic acid and ferulic acid, while caffeic acid was only detected in HP-OCP. Limited research information is available on the inhibitory effects of 4-hydroxybenzoic acid on β-amyloid formation; however, 4-hydroxybenzoic acid exhibited a half maximal inhibitory concentration (IC_50_) of 6.36 µmol/µmol AChE [40]. This phenolic also reduced hydrogen peroxide-induced oxidative stress, which contributes to neuronal cell death and exhibits neuroprotective effects under excitotoxicity [41]. Ferulic acid exhibited different inhibitory effects on β-amyloid aggregations formed by many β-amyloid species, including Aβ_25–35_, Aβ_1–42_, and Aβ_1–40_, as well as destabilizing β-amyloid pre-aggregation [42]. Oral administration of ferulic acid (30 mg/kg/day) improved behavioral impairment and decreased several biomarkers in the β-amyloidogenic pathway in a cerebral amyloid transgenic mouse model [43]. Ferulic acid also showed synergistic effects with octyl gallate, which improved cognitive functions in a transgenic mouse model of Alzheimer’s disease better than using individual compounds [44]. As the least detected phenolic in HP-OCP, caffeic acid (50 mg/kg/day) exhibited neuroprotective effects by improving memory functions and spatial cognition in an Aβ_25-35_-injected Alzheimer’s disease mouse [45]. Unfortunately, information on the hypertensive effect of 4-hydroxybenzoic acid is unavailable; however, ferulic acid (9.5 mg/kg) reduced blood pressure within 2 h after administration in stroke-prone spontaneously hypertensive rats, while a decrease in serum ACE activity was also observed [46]. Similar results were observed with caffeic acid, whereby an oral administration of 10 and 15 mg/kg/day reduced blood pressure and ACE activity in cyclosporine-induced hypertensive rats [47].

As previously stated, safety assessment, including genotoxicity, is one of the critical requirements for the development of functional ingredients; therefore, the genotoxicity of the optimized HEP-OCP using the standard guidelines from the OECD (Ames test) was investigated. The data showed that HEP-OCP did not induce DNA mutations and was genome-safe (Table 8 and Table 9). According to the phytochemical data showing that 4-hydroxybenzoic acid and ferulic acid were the main phytochemicals in HEP-OCP, these two compounds lack genotoxicity potential [48,49]. Interestingly, both compounds act as potential antioxidants [50,51], which might inhibit DNA damage [52]. Indeed, it has been reported that 4-hydroxybenzoic acid and ferulic acid can prevent DNA damage when pBR322 plasmid DNA is used as a testing model, especially ferulic acids (2–32 µg/mL) that protect DNA from ultraviolet light and hydrogen peroxide [32]. This suggests that HEP-OCP might also be employed as an anti-genotoxic agent. Investigating further is worthwhile.

## 5. Conclusions

This study optimized an effective method for extracting TPCs from *H. heteroclita* OCP waste by-products using a straightforward procedure using an environmentally friendly solvent. The optimized extract contained high levels of phytochemicals, including 4-hydroxybenzoic acid and ferulic acid. The extract also exhibited antioxidant activities and health benefits, especially BACE-1 and ACE. These two enzymes are involved in the pathogenesis of Alzheimer’s disease and hypertension, respectively. Our optimized extract showed promise for further testing as an anti-Alzheimer’s disease agent and as a hypertensive agent. Preliminary genotoxicity testing also demonstrated that the extract did not cause DNA alterations when bioactivation was present or absent. Our results demonstrated the socioeconomic significance of *H. heteroclita* OCPs as a valuable extract with advantageous health effects. However, further research is necessary on the isolation and protective effects of animal models against Alzheimer’s disease and hypertension.

## Figures and Tables

**Table 1:** Variable degrees for each factor in Box–Behnken design (BBD).

Coded	Independent Variables	Variable Degree		
		−1	0	+1
$X_{1}$	Ethanol concentration (% *v*/*v*)	50	70	90
$X_{2}$	Temperature (°C)	30	50	70
X_3_	Time (h)	2	4	6

**Table 2:** Box–Behnken design of independent variables derived from Table 1.

Run	Box–Behnken Design of Independent Variables		
	$X_{1}$: Ethanol (% *v*/*v*)	$X_{2}$: Temperature (°C)	$X_{3}$: Time (h)
1	0 (70)	−1 (30)	−1 (2)
2	0 (70)	0 (50)	0 (4)
3	1 (90)	0 (50)	−1 (2)
4	0 (70)	0 (50)	0 (4)
5	0 (70)	1 (70)	1 (6)
6	1 (90)	1 (70)	0 (4)
7	1 (90)	−1 (30)	0 (4)
8	0 (70)	−1 (30)	1 (6)
9	−1 (50)	−1 (30)	0 (4)
10	−1 (50)	0 (50)	1 (6)
11	−1 (50)	1 (70)	0 (4)
12	−1 (50)	0 (50)	−1 (2)
13	0 (70)	0 (50)	0 (4)
14	0 (70)	1 (70)	−1 (2)
15	1 (90)	0 (50)	1 (6)

**Table 3:** Effects of independent variables (ethanol concentration, extraction temperature, and extraction time) and dependent variables (total phenolic contents, TPCs) derived from the Box–Behnken design (BBD) for extraction of *H. heteroclita* oilseed cake powder obtained from heat and enzymatic pretreatment (HEP-OCP).

Run	Box–Behnken Design of Independent Variables			TPCs (mg of GAE/100 g DW)	
	$X_{1}$: Ethanol (% *v*/*v*)	$X_{2}$: Temperature (°C)	$X_{3}$: Time (h)	Experimental	Predicted
1	0 (70)	−1 (30)	−1 (2)	283.55 ± 8.26 ^ef^	265.24
2	0 (70)	0 (50)	0 (4)	292.69 ± 7.13 ^e^	296.39
3	1 (90)	0 (50)	−1 (2)	143.14 ± 6.02 ^hi^	142.14
4	0 (70)	0 (50)	0 (4)	318.05 ± 7.21 ^d^	296.39
5	0 (70)	1 (70)	1 (6)	270.34 ± 18.32 ^f^	288.65
6	1 (90)	1 (70)	0 (4)	223.33 ± 6.37 ^g^	207.60
7	1 (90)	−1 (30)	0 (4)	133.24 ± 13.27 ^i^	152.55
8	0 (70)	−1 (30)	1 (6)	325.99 ± 5.23 ^d^	309.25
9	−1 (50)	−1 (30)	0 (4)	401.60 ± 14.88 ^b^	417.34
10	−1 (50)	0 (50)	1 (6)	374.29 ± 7.11 ^c^	375.28
11	−1 (50)	1 (70)	0 (4)	427.55 ± 14.20 ^a^	408.24
12	−1 (50)	0 (50)	−1 (2)	381.98 ± 10.16 ^c^	384.55
13	0 (70)	0 (50)	0 (4)	278.42 ± 11.37 ^ef^	296.39
14	0 (70)	1 (70)	−1 (2)	315.07 ± 5.42 ^d^	331.80
15	1 (90)	0 (50)	1 (6)	154.84 ± 7.39 ^h^	152.26

Experimental data are shown as the mean ± standard deviation (SD) of triplicate determinations (*n* = 3). Superscript letters indicate significantly different contents of different TPCs in each run condition at *p* < 0.05 using one-way analysis of variance (ANOVA) and Duncan’s multiple comparison tests; predicted TPCs were calculated using Equation (2); GAE: gallic acid equivalence; DW: dry weight.

**Table 4:** Analysis of variance (ANOVA) and estimated regression coefficients for the response surface quadratic model for total phenolic contents (TPCs) extraction from *H. heteroclita* oilseed cake powder obtained from heat and enzymatic pretreatment (HEP-OCP).

Dependent Variables	Source	Sum of Squares	df	Mean Square	F-Value	*p*-Value	Significance
TPCs	Model	1.16 × 10^5^	9	12,859.56	19.53	0.0022	**
	$X_{1}$	1.08 × 10^5^	1	1.08 × 10^5^	164.50	<0.0001	***
	$X_{2}$	1055.63	1	1055.63	1.60	0.2613	
	$X_{3}$	0.3672	1	0.3672	0.0006	0.9821	
	$X_{1}X_{2}$	1028.9	1	1028.9	1.56	0.2666	
	$X_{1}X_{3}$	94.01	1	94.01	0.1428	0.7210	
	$X_{2}X_{3}$	1899.3	1	1899.3	2.88	0.1502	
	$X_{1}^{2}$	1139.32	1	1139.32	1.73	0.2455	
	$X_{2}^{2}$	1145.09	1	1145.09	1.74	0.2444	
	$X_{3}^{2}$	859.91	1	859.91	1.31	0.3049	
	Residual	3292.39	5	658.48			
	Lack of Fit	2486.71	3	828.9	2.06	0.3436	NS
	Pure Error	805.69	2	402.84			
	Cor Total	1.19 × 10^5^	14				
	R^2^	0.9723					
	Adjusted R^2^	0.9226					

Experimental data are shown as an analysis of variance (ANOVA) for the response surface quadratic model. X_1_: ethanol concentration; X_2_: temperature; X_3_: time. ** and *** show significant differences determined using the software Design-Expert. ** Significant at *p* < 0.01; *** Significant at *p* < 0.001; NS: not significant.

**Table 5:** Phenolic profile and total phenolic contents (TPCs) of a fresh sample and *H. heteroclita* oilseed cake powders (OCPs) extracted under optimized extraction conditions (30 °C for 5 h in 50% (*v*/*v*) ethanol).

Samples	Phenolic Profile (mg/100 g DW)			TPCs (mg GAE/100 g DW)
	4-Hydroxybenzoic Acid	Caffeic Acid	Ferulic Acid	
Fresh	727.68 ± 0.58 ^a^	501.72 ± 1.33 ^a^	896.31 ± 5.34 ^a^	43.87 ± 1.92 ^d^
NP-OCP	571.77 ± 27.80 ^b^	ND	580.45 ± 10.88 ^c^	117.00 ± 9.79 ^c^
HP-OCP	550.52 ± 21.59 ^b^	369.89 ± 2.74 ^b^	660.58 ± 8.75 ^b^	138.00 ± 3.81 ^b^
HEP-OCP	397.64 ± 3.78 ^c^	ND	298.79 ± 0.98 ^d^	413.96 ± 12.00 ^a^

Experimental data are shown as the mean ± standard deviation (SD) of triplicate determinations (*n* = 3). Different lowercase letters denote significantly different contents of phenolics at *p* < 0.05 among samples using one-way ANOVA, followed by Duncan’s multiple comparison test. All phenolics detected here are in selective reaction monitoring (SRM) mode with ion mass [M-H]. The parent ion of 4-hydroxybenzoic acid is *m*/*z* 137.05, with SRM transitions of *m*/*z* 92.970, 65.000, and 75.000. The parent ion of caffeic acid is *m*/*z* 179.038 with SRM transitions of *m*/*z* 135.054, 107.071, and 85.042, while the parent ion of ferulic acid is *m*/*z* 179.038 with SRM transitions of *m*/*z* 149.125, 177.970, and 134.042. NP-OCP: OCP obtained from no pretreatment method; HP-OCP: OCP obtained from heat pretreatment by drying at 55 °C until reaching 10% moisture content; HEP-OCP: OCP obtained from heat and enzymatic pretreatment using 2.98% (*w*/*w*) enzyme loading, 48 °C of incubation temperature, and 76 min of incubation time; GAE: gallic acid equivalent; DW: dry weight.

**Table 6:** Antioxidant activities determined by 2,2-diphenyl-1-picrylhydrazyl (DPPH) radical scavenging, ferric ion reducing antioxidant power (FRAP), and oxygen radical absorbance capacity (ORAC) assays of fresh samples and *H. heteroclita* oilseed cake powders (OCPs) extracted under optimized extraction conditions (30 °C for 5 h in 50% (*v*/*v*) ethanol).

Samples	Antioxidant Activities		
	DPPH Radical Scavenging Assay (µmol TE/100 g DW)	FRAP Assay (µmol TE/g DW)	ORAC Assay (µmol TE/g DW)
Fresh	0.04 ± 0.00 ^d^	0.88 ± 0.09 ^d^	9.65 ± 0.91 ^d^
NP-OCP	0.10 ± 0.01 ^c^	2.14 ± 0.14 ^c^	25.32 ± 2.32 ^c^
HP-OCP	0.13 ± 0.01 ^b^	2.85 ± 0.09 ^b^	33.19 ± 2.04 ^b^
HEP-OCP	0.56 ± 0.05 ^a^	7.78 ± 0.56 ^a^	68.72 ± 3.59 ^a^

Experimental data are shown as the mean ± standard deviation (SD) of triplicate determinations (*n* = 3). Different lowercase letters denote significantly different antioxidant activities at *p* < 0.05 among samples using one-way ANOVA, followed by Duncan’s multiple comparison test. NP-OCP: OCP obtained from no pretreatment method; HP-OCP: OCP obtained from heat pretreatment by drying at 55 °C until reaching 10% moisture content; HEP-OCP: OCP obtained from heat and enzymatic pretreatment using 2.98% (*w*/*w*) enzyme loading, 48 °C of incubation temperature, and 76 min of incubation time; TE: Trolox equivalent; DW: dry weight.

**Table 7:** Enzyme inhibitory activities of fresh samples and *H. heteroclita* oilseed cake powders (OCPs) extracted under optimized extraction conditions (30 °C for 5 h in 50% (*v*/*v*) ethanol).

Enzyme Inhibition (% Inhibition)	Samples *			
	Fresh	NP-OCP	HP-OCP	HEP-OCP
Lipase	10.63 ± 0.72	ND	ND	ND
α-Amylase	ND	ND	ND	3.72 ± 0.25
α-Glucosidase	11.56 ± 0.58 ^b^	11.31 ± 0.61 ^b^	9.42 ± 0.85 ^c^	15.81 ± 1.33 ^a^
DPP-IV	ND	ND	ND	16.66 ± 0.76
AChE	ND	ND	ND	ND
BChE	ND	ND	ND	ND
BACE-1	39.80 ± 3.16 ^d^	65.40 ± 2.40 ^c^	71.71 ± 0.92 ^b^	76.60 ± 2.98 ^a^
ACE	25.77 ± 2.11 ^c^	68.47 ± 3.49 ^b^	74.82 ± 2.99 ^b^	93.52 ± 2.90 ^a^

Experimental data are shown as the mean ± standard deviation (SD) of triplicate determinations (*n* = 3). Different lowercase letters denote significantly different enzyme inhibitions at *p* < 0.05 among samples using one-way ANOVA, followed by Duncan’s multiple comparison test. * denotes the final concentration of the extract, which is 20 mg/mL. NP-OCP: OCP obtained from no pretreatment method; HP-OCP: OCP obtained from heat pretreatment by drying at 55 °C until reaching 10% moisture content; HEP-OCP: OCP obtained from heat and enzymatic pretreatment using 2.98% (*w*/*w*) enzyme loading, 48 °C of incubation temperature, and 76 min of incubation time; DPP-IV: dipeptidyl peptidase IV; AChE: acetylcholinesterase; BChE: butyrylcholinesterase; BACE-1: beta-secretase; ACE: angiotensin-converting enzyme; ND: not detected.

**Table 8:** Mutagenicity effects of *H. heteroclita* oilseed cake powders obtained from heat and enzymatic pretreatment (HEP-OCP) extracted under optimized extraction conditions (30 °C for 5 h in 50% (*v*/*v*) ethanol) on five *S. typhimurium* strains without S9 extract (−S9).

Doses (µg/Plate)	TA98		TA100		TA102		TA1535		TA1537	
	Revertant Colonies	MR	Revertant Colonies	MR	Revertant Colonies	MR	Revertant Colonies	MR	Revertant Colonies	MR
Neg	82.50 ± 2.36	1.00 (−)	75.67 ± 2.81	1.00 (−)	371.00 ± 13.33	1.00 (−)	10.17 ± 0.69	1.00 (−)	9.50 ± 0.96	1.00 (−)
10	82.17 ± 1.77	1.00 (−)	75.50 ± 1.89	1.00 (−)	369.67 ± 7.20	1.00 (−)	10.33 ± 0.75	1.02 (−)	9.50 ± 0.76	1.00 (−)
100	82.00 ± 1.63	0.99 (−)	74.83 ± 2.11	0.99 (−)	367.50 ± 12.20	0.99 (−)	9.50 ± 0.50	0.93 (−)	10.17 ± 0.69	1.07 (−)
500	80.83 ± 0.69	0.98 (−)	73.67 ± 1.97	0.97 (−)	365.00 ± 6.93	0.98 (−)	10.33 ± 0.94	0.98 (−)	9.17 ± 0.37	0.96 (−)
1000	81.00 ± 0.82	0.98 (−)	75.33 ± 2.21	1.00 (−)	363.83 ± 9.32	0.98 (−)	10.33 ± 1.11	1.02 (−)	9.33 ± 0.75	0.98 (−)
2000	81.83 ± 1.57	0.99 (−)	74.50 ± 1.71	0.98 (−)	363.00 ± 6.86	0.98 (−)	10.83 ± 0.90	1.07 (−)	9.67 ± 0.75	1.02 (−)
4-NQO	1152.67 ± 32.78	13.97 (+)								
NaN_3_			1060.00 ± 31.58	14.01 (+)			295.00 ± 6.95	29.02 (+)		
MMC					1056.33 ± 30.32	2.86 (+)				
9-AA									788.67 ± 8.06	83.02 (+)

All data are shown as the mean ± standard deviation (SD) of triplicate experiments (*n* = 3). (−) indicates the mutagenicity ratio (MR) of ≤1, and (+) indicates the MR of ≥2. Negative control (Neg) is dimethyl sulfoxide (DMSO) used as a solvent control. Positive controls included 4-nitroquinoline-1-oxide (4NQO), sodium azide (NaN_3_), mitomycin C (MMC), and 9-aminoacridine (9-AA).

**Table 9:** Mutagenicity effects of *H. heteroclita* oilseed cake powders obtained from heat and enzymatic pretreatment (HEP-OCP) extracted under optimized extraction conditions (30 °C for 5 h in 50% (*v*/*v*) ethanol) on five *S. typhimurium* strains with S9 extract (+S9).

Doses (µg/Plate)	TA98		TA100		TA102		TA1535		TA1537	
	Revertant Colonies	MR	Revertant Colonies	MR	Revertant Colonies	MR	Revertant Colonies	MR	Revertant Colonies	MR
Neg	83.83 ± 2.79	1.00 (−)	83.50 ± 2.22	1.00 (−)	359.33 ± 13.83	1.00 (−)	12.50 ± 0.96	1.00 (−)	9.67 ± 0.75	1.00 (−)
10	81.50 ± 1.38	0.97 (−)	81.33 ± 1.49	0.97 (−)	363.83 ± 11.47	1.01 (−)	12.83 ± 1.07	1.03 (−)	10.60 ± 1.26	1.09 (−)
100	82.33 ± 1.60	0.98 (−)	82.67 ± 2.05	0.99 (−)	363.67 ± 9.48	1.01 (−)	12.67 ± 1.37	1.01 (−)	9.67 ± 0.94	1.00 (−)
500	82.83 ± 0.90	0.99 (−)	83.67 ± 1.80	1.00 (−)	365.33 ± 16.70	1.02 (−)	13.00 ± 0.82	1.04 (−)	9.50 ± 0.96	0.98 (−)
1000	82.33 ± 1.11	0.98 (−)	83.83 ± 1.34	1.00 (−)	356.83 ± 6.44	0.99 (−)	12.33 ± 1.25	0.99 (−)	9.83 ± 0.69	1.02 (−)
2000	82.50 ± 1.26	0.98 (−)	84.83 ± 1.77	1.02 (−)	358.67 ± 7.18	1.00 (−)	13.33 ± 0.47	1.07 (−)	9.50 ± 0.76	0.98 (−)
2-AA	1086.67 ± 17.07	12.96 (+)	1113.33 ± 47.31	13.33 (+)	1100.00 ± 48.88	3.06 (+)	299.67 ± 13.03	23.97 (+)	194.33 ± 6.21	20.10 (+)

All data are shown as the mean ± standard deviation (SD) of triplicate experiments (*n* = 3). (−) indicates the mutagenicity ratio (MR) of ≤1, and (+) indicates the MR of ≥2. The negative control (Neg) is dimethyl sulfoxide (DMSO) used as a solvent control, and 2-aminoanthracene (2-AA) was used as a positive control.

## Data Availability

Data are contained within this article and Supplementary Data.

## Supplementary Materials

The following supporting information can be downloaded at: https://www.mdpi.com/article/10.3390/foods12234281/s1, Table S1: Colors and moisture contents of fresh sample and *H. heteroclita* oilseed cake powders (OCPs); Figure S1: The liquid chromatography-electrospray ionization tandem mass spectrometry (LC-ESI-MS/MS) chromatograms of (A) fresh sample, (B) no pretreatment oilseed cake powder (NP-OCP), (C) heat pretreatment oilseed cake powder (HP-OCP), and (D) heat and enzymatic pretreatment (HEP-OCP).

## References

[1] Swargiary A., Brahma D. (2017). Phytochemical Analysis and Antioxidant Activity of Hodgsonia heteroclita (Roxb). Indian. J. Pharm. Sci..

[2] Piseskul J., Suttisansanee U., Chupeerach C., Khemthong C., Thangsiri S., Temviriyanukul P., Sahasakul Y., Santivarangkna C., Chamchan R., Aursalung A. (2023). Optimization of Enzyme-Assisted Mechanical Extraction Process of Hodgsonia heteroclita Oilseeds and Physical, Chemical, and Nutritional Properties of the Oils. Foods.

[3] Khuntaseema B., Jomduang S. (2014). Nutritive Values of Inner Pulp from Ma-king Seed (Hodgsonia heteroclite susp. Indochinensis). Agric. Sci. J..

[4] Kaur N., Chugh V., Gupta A.K. (2014). Essential fatty acids as functional components of foods—A review. J. Food Sci. Technol..

[5] Kremmyda L.S., Tvrzicka E., Stankova B., Zak A. (2011). Fatty acids as biocompounds: Their role in human metabolism, health and disease: A review. part 2: Fatty acid physiological roles and applications in human health and disease. Biomed. Pap. Med. Fac. Univ. Palacky. Olomouc Czech Repub..

[6] Usha T., Goyal A.K., Narzary D., Prakash L., Wadhwa G., Babu D., Shanmugarajan D., Middha S.K. (2018). Identification of bioactive glucose-lowering compounds of methanolic extract of Hodgsonia heteroclita fruit pulp. Front. Biosci.-Landmark.

[7] Muhammad Abdul Kadar N.N., Ahmad F., Teoh S.L., Yahaya M.F. (2021). Caffeic Acid on Metabolic Syndrome: A Review. Molecules.

[8] Magnani C., Isaac V.L.B., Correa M.A., Salgado H.R.N. (2014). Caffeic acid: A review of its potential use in medications and cosmetics. Anal. Methods.

[9] Kakkar S., Bais S. (2014). A Review on Protocatechuic Acid and Its Pharmacological Potential. ISRN Pharmacol..

[10] Srinivasan M., Sudheer A.R., Menon V.P. (2007). Ferulic Acid: Therapeutic potential through its antioxidant property. J. Clin. Biochem. Nutr..

[11] Ye L., Hu P., Feng L.P., Huang L.L., Wang Y., Yan X., Xiong J., Xia H.L. (2022). Protective Effects of Ferulic Acid on Metabolic Syndrome: A Comprehensive Review. Molecules.

[12] Arif T. (2015). Salicylic acid as a peeling agent: A comprehensive review. Clin. Cosmet. Investig. Dermatol..

[13] Chemat F., Vian M.A., Cravotto G. (2012). Green extraction of natural products: Concept and principles. Int. J. Mol. Sci..

[14] Khuri A.I., Mukhopadhyay S. (2010). Response surface methodology. WIREs Comput. Stat..

[15] What is Bioethanol?. https://www.esru.strath.ac.uk/EandE/Web_sites/02-03/biofuels/what_bioethanol.htm.

[16] Chemat F., Abert-Vian M., Fabiano-Tixier A.S., Strube J., Uhlenbrock L., Gunjevic V., Cravotto G. (2019). Green extraction of natural products. Origins, current status, and future challenges. TrAC Trends Anal. Chem..

[17] Zarina Z., Tan S.Y. (2013). Determination of flavonoids in Citrus grandis (Pomelo) peels and their inhibition activity on lipid peroxidation in fish tissue. Int. Food Res. J..

[18] Gertenbach D.D. (2002). Solid–Liquid Extraction Technologies for Manufacturing Nutraceuticals.

[19] Wang L., Cheng L., Liu F., Li T., Yu Z., Xu Y., Yang Y. (2018). Optimization of Ultrasound-Assisted Extraction and Structural Characterization of the Polysaccharide from Pumpkin (*Cucurbita moschata*) Seeds. Molecules.

[20] Ahamad J., Amin S., Mir S.R. (2015). Optimization of ultrasound-assisted extraction of charantin from Momordica charantia fruits using response surface methodology. J. Pharm. Bioallied Sci..

[21] De Wilde W.J.J.O., Duyfjes B.E.E. (2008). Cucurbitaceae.

[22] Chupeerach C., Temviriyanukul P., Thangsiri S., Inthachat W., Sahasakul Y., Aursalung A., Wongchang P., Sangkasa-Ad P., Wongpia A., Polpanit A. (2022). Phenolic Profiles and Bioactivities of Ten Original Lineage Beans in Thailand. Foods.

[23] Sirichai P., Kittibunchakul S., Thangsiri S., On-Nom N., Chupeerach C., Temviriyanukul P., Inthachat W., Nuchuchua O., Aursalung A., Sahasakul Y. (2022). Impact of Drying Processes on Phenolics and In Vitro Health-Related Activities of Indigenous Plants in Thailand. Plants.

[24] Luu L.K., Thangsiri S., Sahasakul Y., Aursalung A., Inthachat W., Temviriyanukul P., On-Nom N., Chupeerach C., Suttisansanee U. (2023). Nutrients, Phytochemicals and In Vitro Disease Prevention of Nephelium hypoleucum Kurz Fruit. Nutrients.

[25] Wannasaksri W., On-Nom N., Chupeerach C., Temviriyanukul P., Charoenkiatkul S., Suttisansanee U. (2021). In Vitro Phytotherapeutic Properties of Aqueous Extracted Adenia viridiflora Craib. towards Civilization Diseases. Molecules.

[26] Alara O.R., Abdurahman N.H., Ukaegbu C.I. (2021). Extraction of phenolic compounds: A review. Curr. Res. Food Sci..

[27] Zalazar-García D., Torres E., Rodriguez-Ortiz L., Deng Y., Soria J., Bucalá V., Rodriguez R., Mazza G. (2020). Cleaner and sustainable processes for extracting phenolic compounds from bio-waste. J. Environ. Manag..

[28] Wang C., Li Z., Xiang J., Johnson J.B., Zheng B., Luo L., Beta T. (2023). From Foxtail Millet Husk (Waste) to Bioactive Phenolic Extracts Using Deep Eutectic Solvent Extraction and Evaluation of Antioxidant, Acetylcholinesterase, and α-Glucosidase Inhibitory Activities. Foods.

[29] Musa K.H., Abdullah A., Jusoh K., Subramaniam V. (2011). Antioxidant Activity of Pink-Flesh Guava (Psidium guajava L.): Effect of Extraction Techniques and Solvents. Food Anal. Methods.

[30] Sarkis J.R., Michel I., Tessaro I.C., Marczak L.D.F. (2014). Optimization of phenolics extraction from sesame seed cake. Sep. Purif. Technol..

[31] Mansour R.B., Falleh H., Hammami M., Barros L., Petropoulos S.A., Tarchoun N., Ksouri R. (2023). The Use of Response Surface Methodology to Optimize Assisted Extraction of Bioactive Compounds from Cucurbita maxima Fruit By-Products. Processes.

[32] Sevgi K., Tepe B., Sarikurkcu C. (2015). Antioxidant and DNA damage protection potentials of selected phenolic acids. Food Chem. Toxicol..

[33] Narzary D., Middha S.K., Usha T., Brahma B.K., Goyal A.K. (2015). Comparative evaluation of phytochemical constituents of rind, pulp, and seed of Hodgsonia heteroclita fruit encountered in Kokrajhar District, BTAD, Assam, India. World J. Pharma. Res..

[34] Terpinc P., Čeh B., Ulrih N.P., Abramovič H. (2012). Studies of the correlation between antioxidant properties and the total phenolic content of different oil cake extracts. Ind. Crops Prod..

[35] Kaur M., Singh B., Kaur A., Singh N. (2021). Proximate, mineral, amino acid composition, phenolic profile, antioxidant and functional properties of oilseed cakes. Int. J. Food Sci. Technol..

[36] Teh S.S., Bekhit Ael D., Birch J. (2014). Antioxidative Polyphenols from Defatted Oilseed Cakes: Effect of Solvents. Antioxidants.

[37] Barta J., Bartova V., Jarosova M., Svajner J., Smetana P., Kadlec J., Filip V., Kyselka J., Bercikova M., Zdrahal Z. (2021). Oilseed Cake Flour Composition, Functional Properties and Antioxidant Potential as Effects of Sieving and Species Differences. Foods.

[38] Švarc-Gajić J., Rodrigues F., Moreira M.M., Delerue-Matos C., Morais S., Dorosh O., Silva A.M., Bassani A., Dzedik V., Spigno G. (2022). Chemical composition and bioactivity of oilseed cake extracts obtained by subcritical and modified subcritical water. Bioresour. Bioprocess..

[39] Sahasakul Y., Aursalung A., Thangsiri S., Temviriyanukul P., Inthachat W., Pongwichian P., Sasithorn K., Suttisansanee U. (2023). Nutritional Compositions, Phenolic Contents and Antioxidant Activities of Rainfed Rice Grown in Different Degrees of Soil Salinity. Foods.

[40] Budryn G., Majak I., Grzelczyk J., Szwajgier D., Rodríguez-Martínez A., Pérez-Sánchez H. (2022). Hydroxybenzoic Acids as Acetylcholinesterase Inhibitors: Calorimetric and Docking Simulation Studies. Nutrients.

[41] Winter A.N., Brenner M.C., Punessen N., Snodgrass M., Byars C., Arora Y., Linseman D.A. (2017). Comparison of the Neuroprotective and Anti-Inflammatory Effects of the Anthocyanin Metabolites, Protocatechuic Acid and 4-Hydroxybenzoic Acid. Oxid. Med. Cell. Longev..

[42] Kikugawa M., Tsutsuki H., Ida T., Nakajima H., Ihara H., Sakamoto T. (2016). Water-soluble ferulic acid derivatives improve amyloid-β-induced neuronal cell death and dysmnesia through inhibition of amyloid-β aggregation. Biosci. Biotechnol. Biochem..

[43] Mori T., Koyama N., Guillot-Sestier M.V., Tan J., Town T. (2013). Ferulic acid is a nutraceutical β-secretase modulator that improves behavioral impairment and alzheimer-like pathology in transgenic mice. PLoS ONE.

[44] Mori T., Koyama N., Tan J., Segawa T., Maeda M., Town T. (2017). Combination therapy with octyl gallate and ferulic acid improves cognition and neurodegeneration in a transgenic mouse model of Alzheimer’s disease. J. Biol. Chem..

[45] Kim J.H., Wang Q., Choi J.M., Lee S., Cho E.J. (2015). Protective role of caffeic acid in an Aβ_25-35_-induced Alzheimer’s disease model. Nutr. Res. Pract..

[46] Ardiansyah, Ohsaki Y., Shirakawa H., Koseki T., Komai M. (2008). Novel effects of a single administration of ferulic acid on the regulation of blood pressure and the hepatic lipid metabolic profile in stroke-prone spontaneously hypertensive rats. J. Agric. Food Chem..

[47] Agunloye O.M., Oboh G., Ademiluyi A.O., Ademosun A.O., Akindahunsi A.A., Oyagbemi A.A., Omobowale T.O., Ajibade T.O., Adedapo A.A. (2019). Cardio-protective and antioxidant properties of caffeic acid and chlorogenic acid: Mechanistic role of angiotensin converting enzyme, cholinesterase and arginase activities in cyclosporine induced hypertensive rats. Biomed. Pharmacother..

[48] Maistro E.L., Angeli J.P., Andrade S.F., Mantovani M.S. (2011). In vitro genotoxicity assessment of caffeic, cinnamic and ferulic acids. Genet. Mol. Res..

[49] OECD SIDS Initial Assessment Profile. https://hpvchemicals.oecd.org/UI/handler.axd?id=cd93235e-9715-4766-a0bc-4b219347aef2.

[50] Piazzon A., Vrhovsek U., Masuero D., Mattivi F., Mandoj F., Nardini M. (2012). Antioxidant Activity of Phenolic Acids and Their Metabolites: Synthesis and Antioxidant Properties of the Sulfate Derivatives of Ferulic and Caffeic Acids and of the Acyl Glucuronide of Ferulic Acid. J. Agric. Food Chem..

[51] Velika B., Kron I. (2012). Antioxidant properties of benzoic acid derivatives against Superoxide radical. Free Radic. Antioxid..

[52] Kaur P., Purewal S.S., Sandhu K.S., Kaur M. (2019). DNA damage protection: An excellent application of bioactive compounds. Bioresour. Bioprocess..

