# Supplementary material for: Green Extraction of Hodgsonia heteroclita Oilseed Cake Powder to Obtain Optimal Antioxidants and Health Benefits
Source: Foods. 2023 Nov 27;12(23):4281. doi: 10.3390/foods12234281 (PMC10706092; doi:10.3390/foods12234281)

Supplementary materials

Green Extraction of *Hodgsonia heteroclita* Oilseed Cake Powder to Obtain Optimal Antioxidants and Health Benefits

Woorawee Inthachat, Sirinapa Thangsiri, Chanakan Khemthong, Nattira On-Nom, Chaowanee Chupeerach, Yuraporn Sahasakul, Piya Temviriyanukul † and Uthaiwan Suttisansanee *,†

Food and Nutrition Academic and Research Cluster, Institute of Nutrition, Mahidol University, Salaya, Phuttamonthon, Nakhon Pathom 73170, Thailand; woorawee.int@mahidol.ac.th (W.I.); sirinapa.tha@mahidol.ac.th (S.T.); chanakan.khe@mahidol.ac.th (C.K.); nattira.onn@mahidol.ac.th (N.O.-N.); chaowanee.chu@mahidol.ac.th (C.C.); yuraporn.sah@mahidol.edu (Y.S.); piya.tem@mahidol.ac.th (P.T.)

* Correspondence: uthaiwan.sut@mahidol.ac.th

† These authors contributed equally to this work

Supplementary Table S1:

Colors and moisture contents of fresh sample and *H. heteroclita* oilseed cake powders (OCPs).

Samples	Color Analysis			Moisture Content (%)
	L*	a*	b*	
Fresh	73.43 ± 0.22	0.35 ± 0.04	20.10 ± 0.06	2.82 ± 0.15
NP-OCP	75.83 ± 1.92	2.63 ± 0.06	22.30 ± 0.10	3.68 ± 0.52
HP-OCP	80.13 ± 1.13	1.88 ± 0.48	21.17 ± 1.28	3.64 ± 0.63
HEP-OCP	65.41 ± 1.38	11.20 ± 0.68	35.95 ± 1.22	4.68 ± 0.52

All data are expressed as mean ± standard deviation (SD) of triplicate experiments (*n* = 3). Colors were analyzed by a ColorFlex EZ spectrophotometer, where L* representing dark (0) to white (100) colors, a* representing green (-) to red (+) colors, and b* representing blue (-) to yellow (+) colors. NP-OCP: OCP obtained from no pretreatment method; HP-OCP: OCP obtained from heat pretreatment by drying at 55 °C until reaching 10% moisture content; HEP-OCP: OCP obtained from heat and enzymatic pretreatments using 2.98% (*w*/*w*) enzyme loading, 48 °C of incubation temperature and 76 min of incubation time.

Supplementary Figure S1:

The liquid chromatography-electrospray ionization tandem mass spectrometry (LC-ESI-MS/MS) chromatograms of (A) fresh sample, (B) no pretreatment oilseed cake powder (NP-OCP), (C) heat pretreatment oilseed cake powder (HP-OCP) and (D) heat and enzymatic pretreatment (HEP-OCP).

A

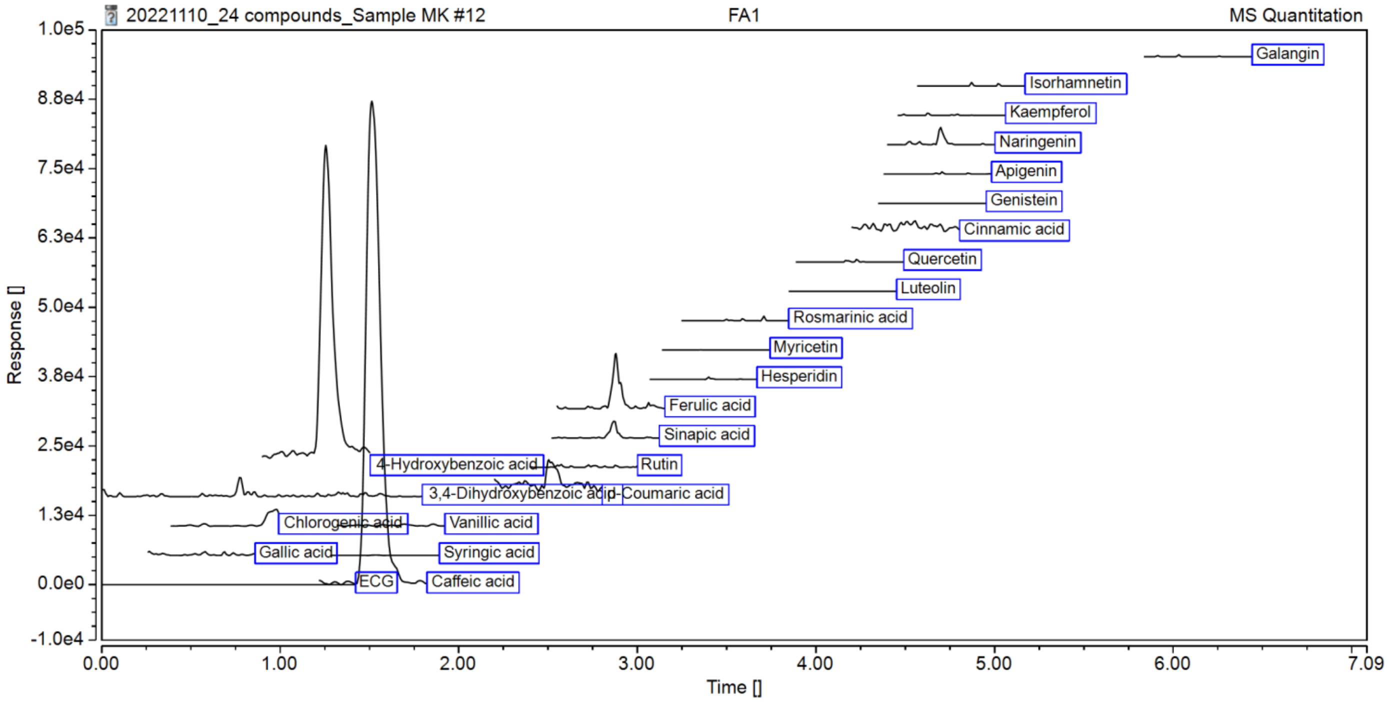

B

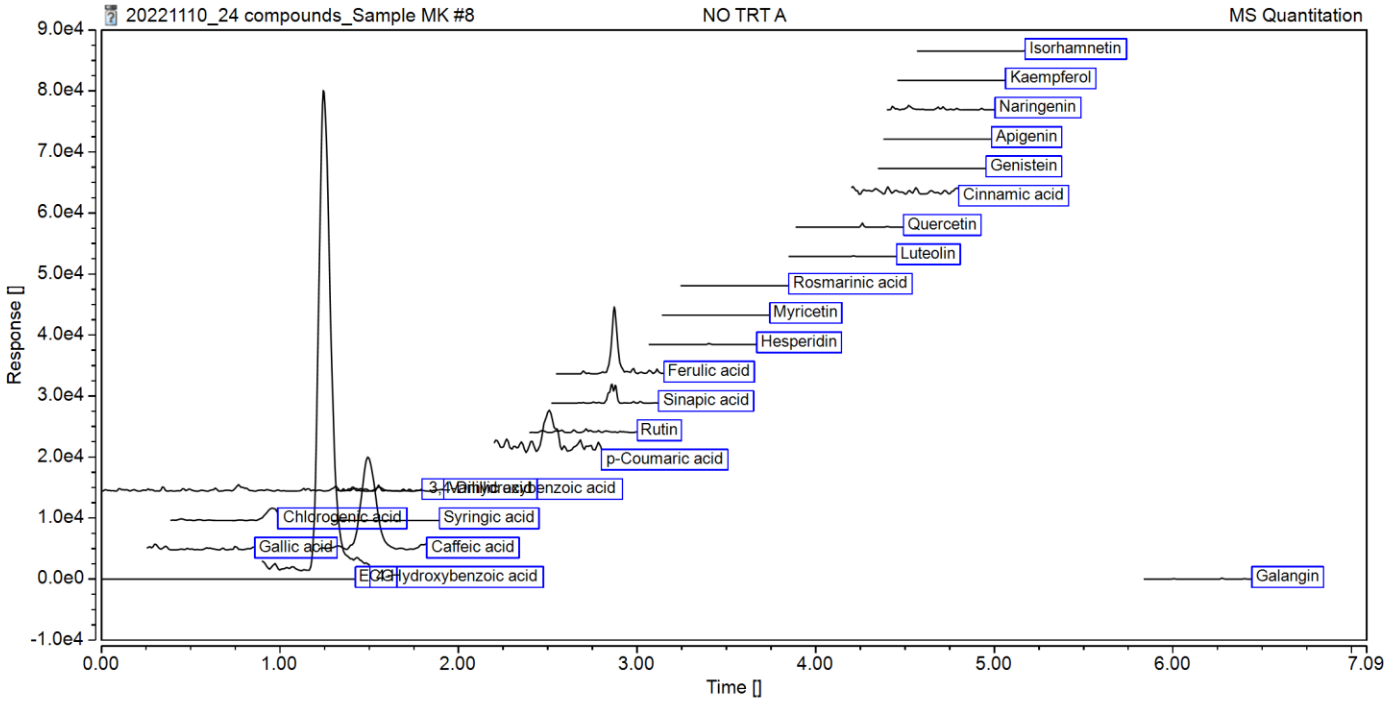

Supplementary Figure S1 (Cont.):

The liquid chromatography-electrospray ionization tandem mass spectrometry (LC-ESI-MS/MS) chromatograms of (A) fresh sample, (B) no pretreatment oilseed cake powder (NP-OCP), (C) heat pretreatment oilseed cake powder (HP-OCP) and (D) heat and enzymatic pretreatment (HEP-OCP).

C

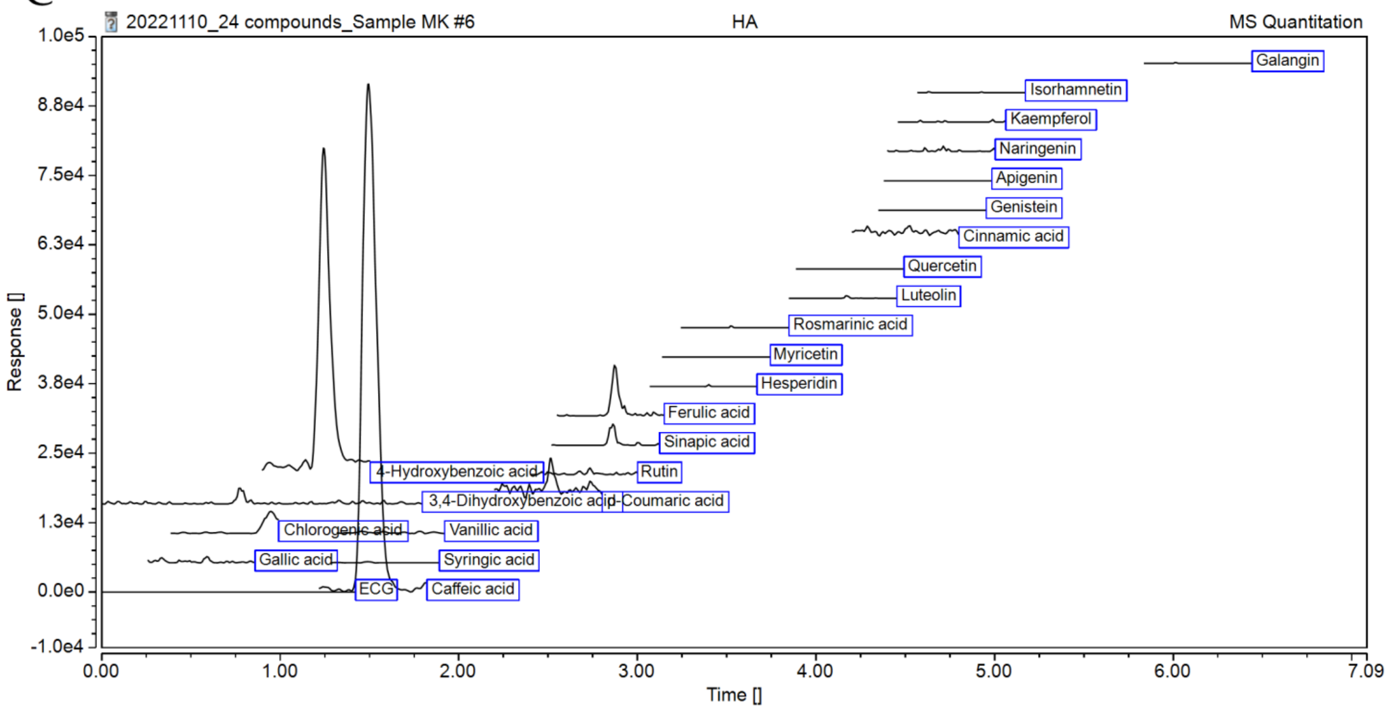

D

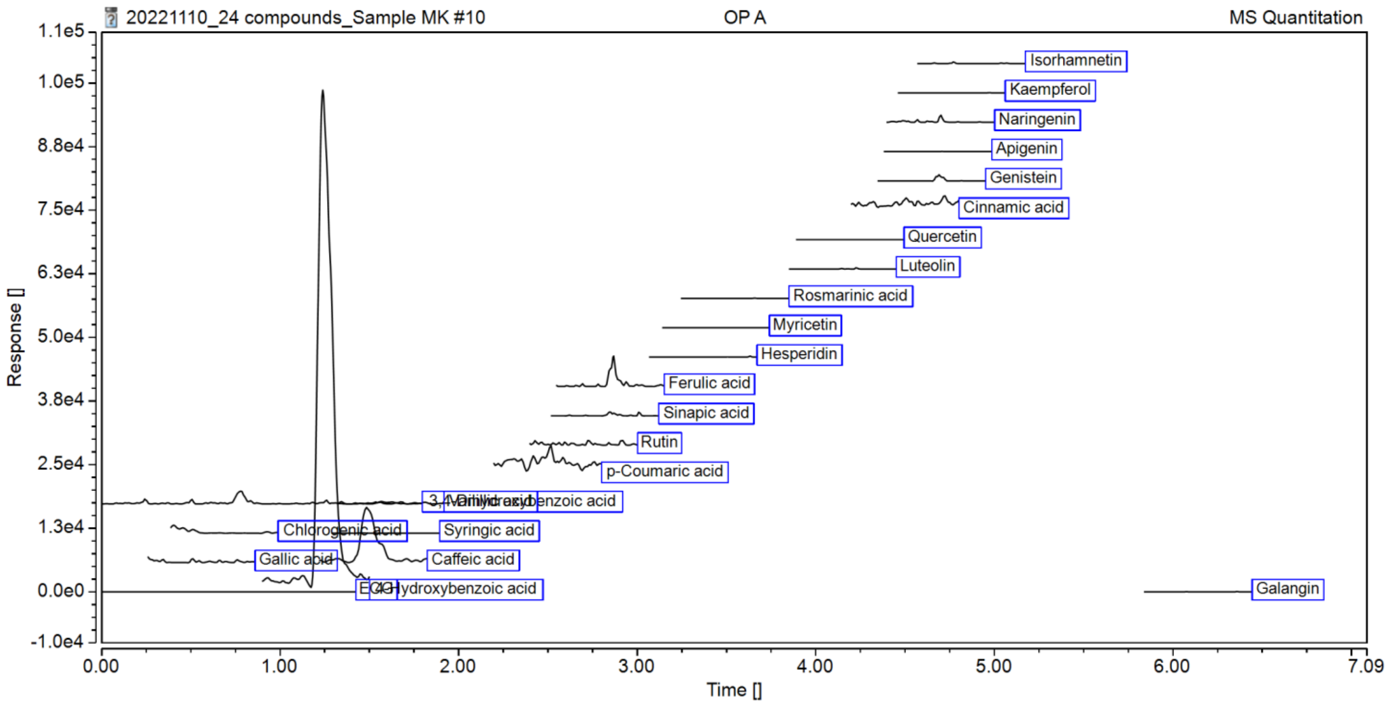

Supplement: Supplementary file 1 [file foods-12-04281-s001.zip › foods-2711061-supplementary.pdf]
